# Supplementary figures and images for: Dietary Supplementation with Lysozyme–Cinnamaldehyde Conjugates Enhances Feed Conversion Efficiency by Improving Intestinal Health and Modulating the Gut Microbiota in Weaned Piglets Infected with Enterotoxigenic Escherichia coli
Source: Animals (Basel). 2023 Nov 13;13(22):3497. doi: 10.3390/ani13223497 (PMC10668808; doi:10.3390/ani13223497)

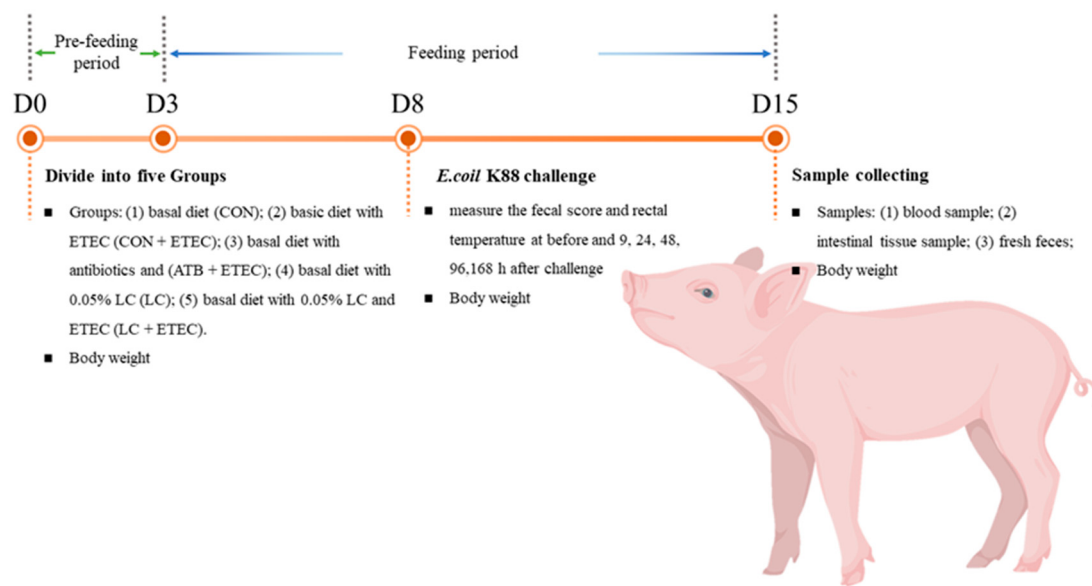

Figure S1. the animal experiment design

Supplement: Supplementary file 1 [file animals-13-03497-s001.zip › Figure S1.pdf]

Original picture of protein band

Duodenum

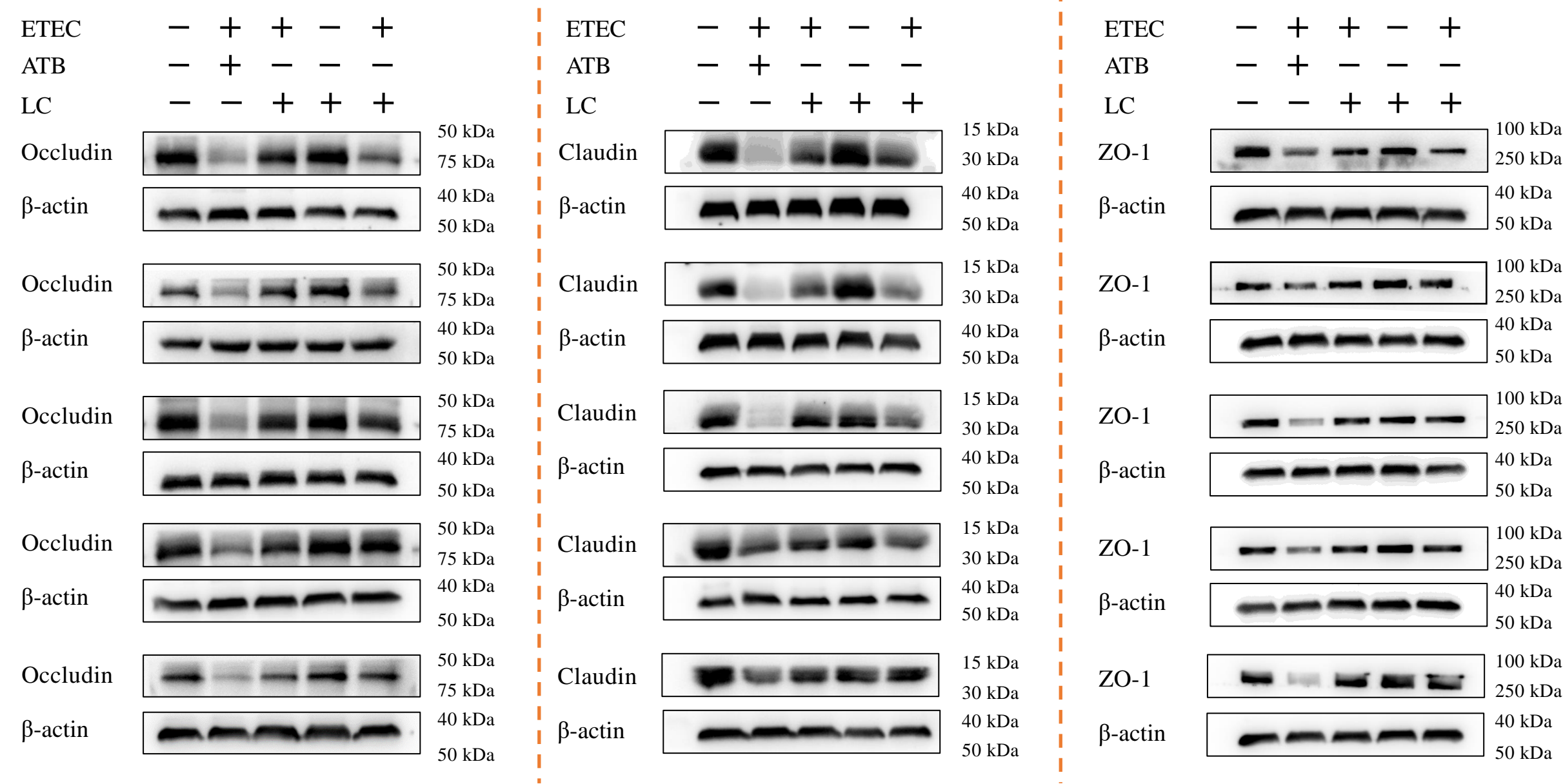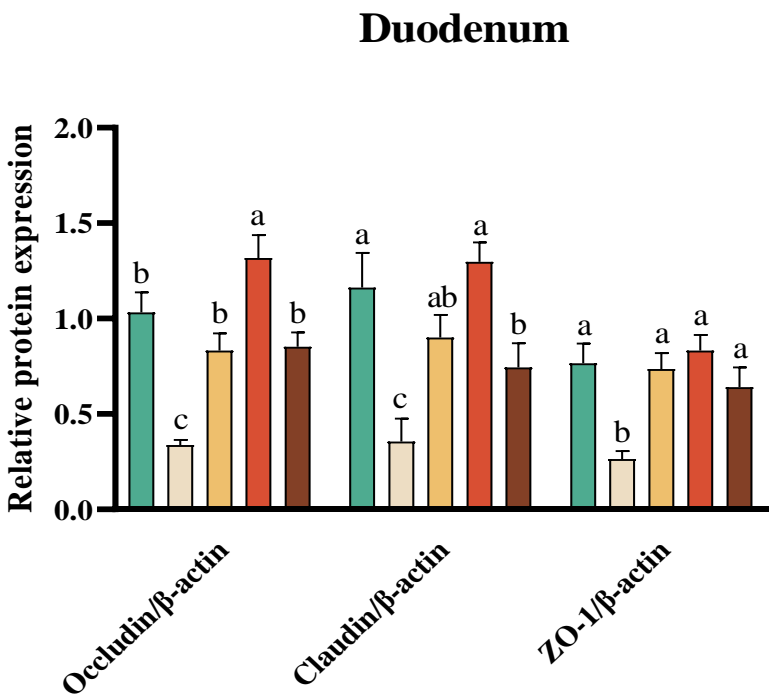

Jejunum

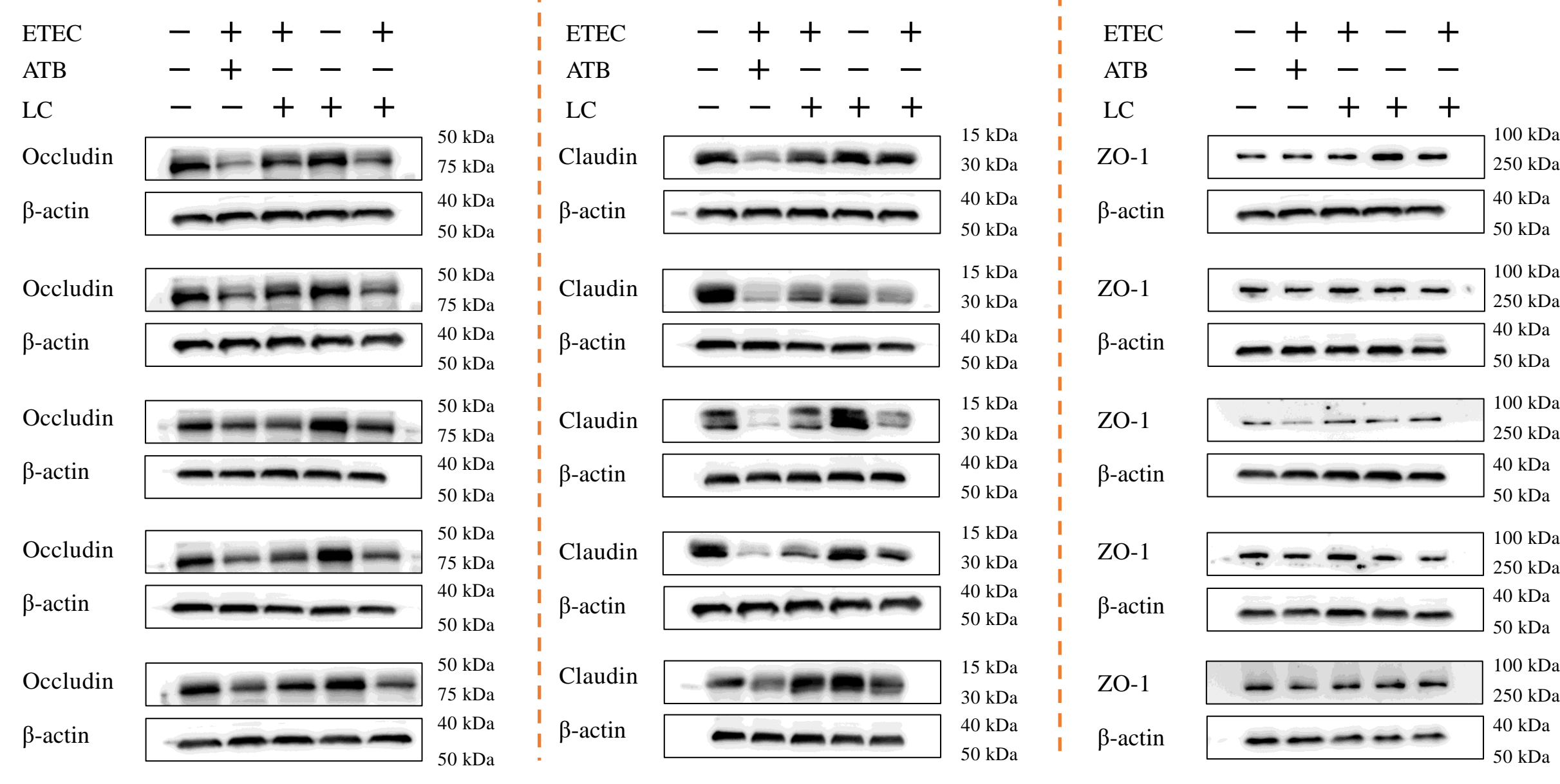

Supplement: Supplementary file 1 [file animals-13-03497-s001.zip › Figure S2.pdf]
